# Diagnostic, clinical management, and outcomes in patients with spinal dural arteriovenous fistula

**DOI:** 10.3389/fsurg.2024.1374321

**Published:** 2024-03-05

**Authors:** Andreas Filis, Sergio M. F. Romualdo, Kay Engellandt, Ibrahim El-Battrawy, Dino Podlesek, Tareq A. Juratli, Ilker Y. Eyüpoglu, Gabriele Schackert, Mido Max Hijazi

**Affiliations:** ^1^ Department of Neurosurgery, Technische Universität Dresden, Faculty of Medicine, and University Hospital Carl Gustav Carus, Dresden, Germany; ^2^ Institute of Diagnostic and Interventional Neuroradiology, Technische Universität Dresden, Faculty of Medicine, and University Hospital Carl Gustav Carus, Dresden, Germany; ^3^ Department of Cardiology, Bergmannsheil University Hospital, Ruhr University Bochum, Bochum, Germany

**Keywords:** spinal arteriovenous malformation, spinal dural fistula, surgical treatment, SDAVF, spinal angiography, spinal cord edema

## Abstract

**Background:**

Spinal dural arteriovenous fistulas (SDAVFs) are rare spinal vascular malformations, but account for 70 to 80% of all spinal arteriovenous malformations. SDAVFs can be treated either surgically or endovascularly, with surgical treatment appearing to lead to higher closure rates. Our aim was to analyze the demographic data, diagnostic history, treatment characteristics and clinical short- and long-term outcomes.

**Methods:**

The medical records of 81 patients who underwent surgical (*n* = 70, 86.4%) and endovascular (*n* = 11, 13.6%) treatment for SDAVF at a university hospital between 2002 and 2023 were retrospectively analyzed.

**Results:**

SDAVF was observed more frequently in men than women (61, 75.3% vs. 20, 24.7%) with a mean age of 63.5 ± 12.7 years and a mean duration of symptoms to diagnosis of 12.0 ± 12.8 months. The most common first symptom was gait disturbance (36, 44.4%), followed by sensory disturbance (24, 29.6%). The location of the fistula point was most common in the lower thoracic region (36, 44.5%), followed by the lumbar region (23, 28.4%). Incomplete or failed occlusion of the fistula occurred in 8 patients (9.9%), with 6 patients (7.4%) undergoing further treatment either surgically or endovascularly. Treatment- or hospital-related complications were observed in 16 patients (19.8%). A single-level laminectomy was the most common approach (31, 44.3%), followed by single-level hemilaminectomy (28, 40.0%), and unilateral interlaminar fenestration (11, 15.7%). Back pain or radiculopathy was observed in 58% of patients (47/81) pre-treatment and had already decreased to 24.7% at hospital discharge (*p* < 0.001). No significant differences were observed in sensory disturbances (*p* = 0.681). The median of American Spinal Injury Association motor score (ASIA-MS) was 94 [82.5–100] at admission, 98 [86.5–100] at hospital discharge, 100 [90–100] at the first, second, and third follow-up (*p* = 0.019). The median modified Aminoff-Logue scale (mALS) was 5 [2–7] at admission, 3 [1–6] at hospital discharge, 2 [1–5] at the first follow-up, 2 [0.5–5] at the second follow-up and 2 [1–7] at the third follow-up (*p* = 0.006).

**Conclusions:**

SDAVF occurs predominantly in men in the 6th decade of life and can be safely and effectively treated surgically and endovascularly, improving symptoms such as pain and motor deficits, gait disturbances as well as bowel and bladder dysfunction, but not sensory disturbances.

## 1 Introduction

Spinal dural arteriovenous fistulas (SDAVFs) result from an abnormal arteriovenous shunt between a radicular artery and vein dorsal to the dura sleeve surface and without an intervening capillary network, causing retrograde drainage into the coronal venous plexus of the spinal cord (1–6). This leads to venous hypertension and reduces the spinal cord perfusion, causing ischemia and oedema (1). The pathophysiologic nature of the associated myelopathy in SDAVF was described by Aminoff and Logue in 1974 (7).

SDAVFs represent a rare entity with a diverse and often misleading clinical presentation and account for the majority of spinal vascular malformations (∼70%) (8). DAVF predominantly affects men (80%) in their fifth or sixth decade (6). The fistula point of SDAVFs most commonly arises from the lower thoracic and upper lumbar vertebral segmental arteries between T6 and L2 (6). Many studies have documented a predominance on the left side (9, 10).

Spinal digital subtraction angiography (DSA) is considered to be the gold standard for the diagnosis of SDAVF (6). The development of high-resolution DSA has improved the assessment of the exact location of SDAVF fistulous point (11–14). The aim of treatment is to interrupt the fistulous arterial and venous point (2, 12, 13, 15, 16). Even though surgery represents the gold standard of treatment, endovascular treatment has become an essential alternative treatment option (17, 18).

Symptoms include a combination of lower limb weakness, gait disturbances, pain, sensory symptoms (paraesthesia, hypesthesia, anaesthesia, or hyperesthesia), bowel and bladder dysfunction (6). Symptoms are usually progressive, with a gradual worsening after the onset of symptoms over a period of 6 months to 2 years, although rapid deterioration has also been reported (19).

The Diagnosis is often difficult to find. Donghai et al. reported about 265 misdiagnosed cases in cohort of 326 patients, 120 had degenerative disc disease, and 62 were treated incorrectly prior to the diagnosis of SDAVF (20). Previous studies on SDAVFs concentrated mainly on outcome comparisons between endovascular and surgical treatment (21). Only a few studies reported on neurological status pre- and post-treatment of SDAVFs. Therefore, we investigated patients’ pre-treatment baseline characteristics, diagnostic history, and neurological outcomes over a one-year post-treatment period.

## 2 Materials and methods

### 2.1 Study design

We conducted a retrospective observational study to evaluate patients who underwent surgery or endovascular treatment at our neurosurgical-neuroradiological university centre in Dresden between 2002 and 2023 and suffered from SDAVF. Eighty-one patients with SDAVF were identified, of whom seventy were treated surgically and eleven endovascularly.

**Figure 1:**
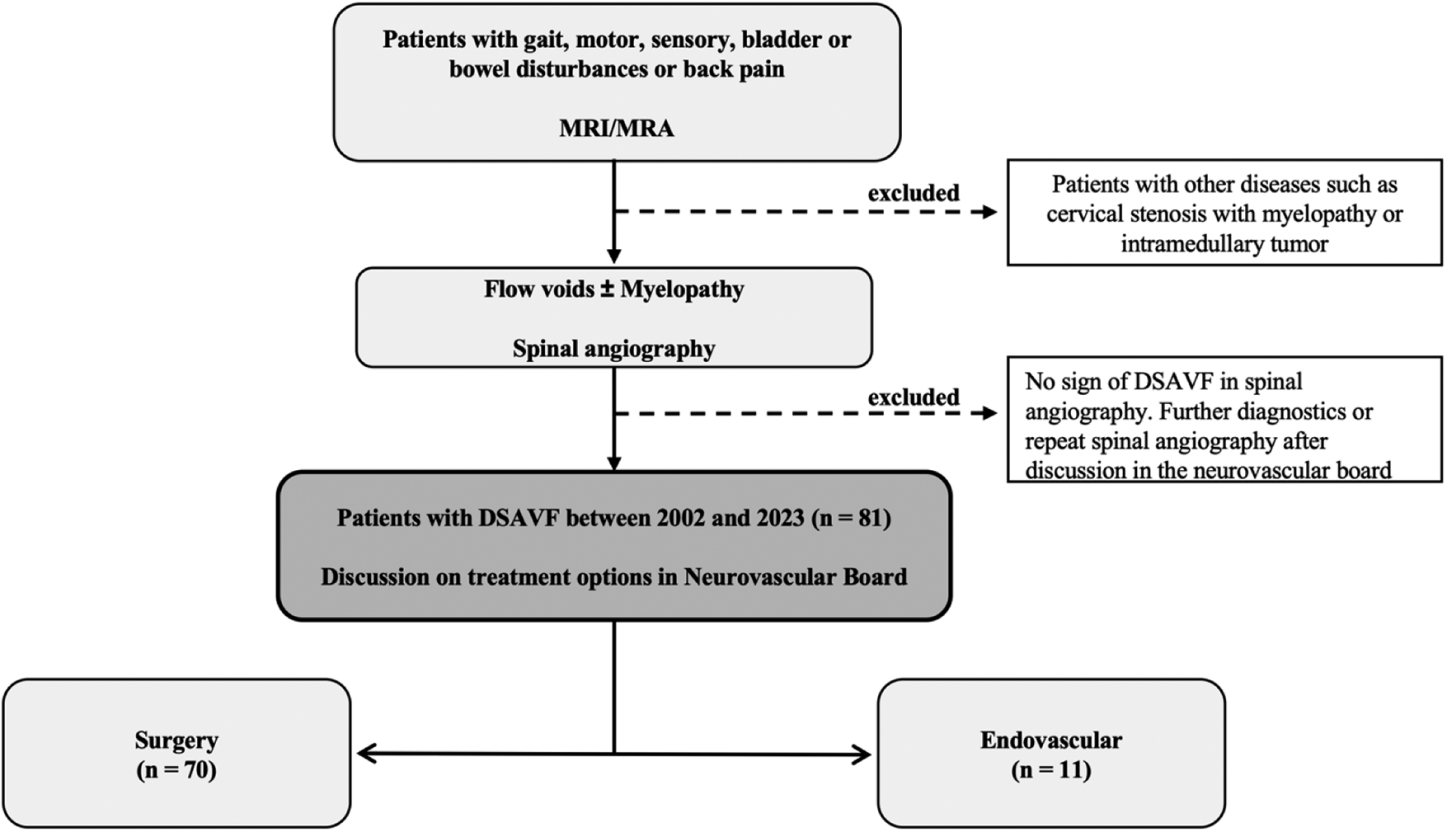
Flow charts. This figure shows our flow charts for diagnosis of spinal dural arteriovenous fistula (SDAVF). MRI, magnetic resonance imaging; MRA, magnetic resonance angiography; DSA, digital subtraction angiography.

Patients with suspected SDAVF on MRI/MRA (vascular myelopathy and/or flow voids) and corresponding symptoms (disturbances of gait, sensory function, motor function, bowel or bladder function or pain) were included in the study. Patients without evidence of SDAVF in the DSA were excluded from the study (Figure 1).

### 2.2 Patient data

The study was approved by our local ethics committee (Reference number BO-EK-437102023). After case identification, patients’ data were collected via the ORBIS system (ORBIS, Dedalus, Bonn, Germany) and neuroimaging studies through the IMPAX system (IMPAX, Impax Asset Management Group plc, London, UK). Radiological data, including MRI, MRA, and DSA of the spine were available for review.

Data collected included age, gender, duration of symptoms to diagnosis, time from diagnosis to surgery or intervention, history of comorbidity (vascular disease, coronary heart disease, stroke, hypertension, hyperlipidaemia, degenerative spine disease, vertebral fractures, rheumatic diseases, drugs that trigger bleeding, use of corticosteroids, and BMI), performance of a preoperative or postoperative MRI/MRA/DSA, number of incomplete or failed occlusions, number of secondary treatments performed (surgery or embolization), recording of treatment- or hospital-related complications, side of the fistula, location of the fistula site, type of surgical approach, duration of surgery, surgical strategy in the form of coagulation with transection or combined (clipping, coagulation, ligation, transection and clip removal), use of indocyanine green fluorescein angiography (ICGFA), use of intraoperative microvascular doppler sonography (IOMVDS), type of embolization materials (Histoacryl, Squid, Onyx), first symptom, neurological status pre-treatment, at the time of hospital discharge, first follow-up (3 months after discharge), second follow-up (6 months after discharge), third follow-up (12 months after discharge), American Spinal Injury Association motor score (ASIA-MS), and modified Aminoff-Logue scale of disability (mALS) (22).

### 2.3 Clinical management

Clinical symptoms such as pain, disturbances of gait, sensory function, motor function, bowel or bladder function associated with MRI/MRA signs of myelopathy and flow voids form the basis for the suspected diagnosis of SDAVF. The diagnosis is confirmed by digital subtraction angiography (DSA). Each case was discussed in a multidisciplinary board with neurointerventional radiologists and neurosurgeons. If two therapeutic options were considered, the patient was usually informed and educated about both treatment options. The decision on the procedure was left to the patient. Until 2012, endovascular treatment was preferred in our hospital as a less invasive procedure. At that time, surgical treatment was suggested if endovascular treatment failed or was not feasible (vertebral artery or adamkiewicz proximity with inadvertent risk of embolization). Since 2012, surgical treatment became the treatment of choice in our hospital and endovascular therapy was considered as an alternative. In all cases, DSA and MRI/MRA were carefully reviewed by the neurosurgeon and a neurointerventional radiologist prior to surgery to determine the exact location and side of the fistula.

The location of the fistula was precisely determined using conventional fluoroscopy prior to the surgical procedure. The patient is carefully positioned in a prone position. In high- or mid-thoracic DSAVF cases, radiopaque markers are useful for intraoperative identification of the target level. The procedure was achieved through a single-level hemilaminectomy or laminectomy. A single-level laminectomy allows for easier watertight closure of the dura, especially in older and overweight patients, but may result in spinal instability, although the facet joint is not removed. A median skin incision of 3 cm is made, followed by uni- or bilateral tendon muscle disinsertion to expose the targeted hemi-lamina or lamina, which is then carefully resected with a diamond drill or/and a Kerrison bone punch. In rare cases, a unilateral interlaminar fenestration is performed, here the median skin incision is 2 cm, and a unilateral tendon muscle dissection is performed to expose an interlaminar window. Fluoroscopic reconfirmation of the level is required before opening the dura. The upper hemilamina is narrowed with the bone punch or diamond drill without damaging the facet joints. The lower part of the upper pedicle including the target nerve root with the accompanying artery can be seen here. The bones are also narrowed on the contralateral side in order to see the midline of the dura and later to facilitate the watertight dural sutures. The flavum is now resected and the dura exposed.

Under microscopic magnification, the dura mater is opened along the midline. The arachnoid is left intact, and the dura tack-up sutures are placed to retract the dural edges to minimize epidural bleeding and increase the view field. The arachnoid is then opened and the arterialized vein is identified as it exits the dura and then dissected free of arachnoid adhesions and the adjacent nerve roots. The fistula is then clipped with a temporary aneurysm clip and a change in the colour and turgor of the distal part of the clipped arterialized vein can be seen immediately. Intraoperative ICGFA and/or IOMVDS can be performed to confirm closure of the fistula. The distal and proximal arterialized vein is coagulated, ligated on both sides, sharply incised and then the temporary clip is removed. As an alternative method, the fistula is coagulated and sharply divided. After removal of the clip, ICGFA and/or IOMVDS were repeated to assess complete closure. A watertight dural suture was created and then fibrin sealant or biological glue was used. No drainage was placed in either surgical technique.

A postoperative spinal DSA or MRI/MRA was performed within the first 3 days after treatment to assess fistula closure and any postoperative complications. MRI/MRA was performed 3, 6 and 12 months after surgical or endovascular treatment to assess the disappearance of myelopathy and the regression of abnormal flow voids.

### 2.4 Statistical analysis

All statistical analyses were carried out using the software package SPSS (SPSS Statistics 28, IBM, Armonk, New York, USA). Following the initial descriptive analysis of the variables, Kruskal-Wallis tests were used to determine statistically significant differences (*p* < 0.05) as a non-parametric method for comparing three or more variables (pre-treatment, at discharge, at first, second and third follow-up). Categorical variables were compared by the Chi-squared or Fisher exact tests, as appropriate. All statistical tests were two-tailed, and a *p*-value *p* < 0.05 was considered statistically significant.

## 3 Results

### 3.1 Baseline characteristics of SDAVF patients

#### 3.1.1 General features

A total of 81 patients with SDAVF were diagnosed and treated either surgically or endovascularly at our institution between 2002 and 2023. Of these, 61 were men (75.3%) and 20 women (24.7%) with an average age of 63.5 ± 12.7 years. The mean duration of symptoms until diagnosis was 12.0 ± 12.8 months. In 24 of the 81 patients (29.6%) the symptoms were shorter than 3 months, while in 57 patients (70.4%) they were longer than 3 months. The mean time from suspected diagnosis on MRI/MRA to surgical or endovascular treatment was 24.4 ± 25 days. The baseline data are summarized in Table 1.

**Table 1:** Baseline characteristics of SDAVF patients.

General features	
Age, mean ± SD	63.5 ± 12.7
Female gender, *n* (%)	20 (24.7%)
Symptom duration to diagnosis, mean ± SD (months)	12.0 ± 12.8
Acute symptoms progression (<3 months), *n* (%)	24 (29.6%)
Chronic symptoms progression (>3 months), *n* (%)	57 (70.4%)
Time from diagnosis to surgery or intervention, mean ± SD (days)	24.4 ± 25
Comorbidity	
Vascular disease, coronary heart disease, or stroke, *n* (%)	31 (38.3%)
Hypertension, *n* (%)	57 (70.4%)
Hyperlipidemia, *n* (%)	23 (28.4%)
Degenerative spine disease or vertebral fracture, *n* (%)	29 (35.8%)
Rheumatic disorders or autoimmune disease, *n* (%)	1 (1.2%)
Drugs causing bleeding, *n* (%)	21 (25.9%)
Use of corticosteroids, *n* (%)	15 (18.5%)
Obesity (BMI > 30 kg/m^2^), *n* (%)	19 (23.5%)
Diagnostics	
Preoperative spinal MRI/MRA, *n* (%)	81 (100.0%)
Preoperative spinal DSA, *n* (%)	81 (100.0%)
Postoperative spinal MRI/MRA, *n* (%)	65 (80.2%)
Postoperative spinal DSA, *n* (%)	63 (77.8%)
Treatments	
Embolization, *n* (%)	11 (13.6%)
Surgery, *n* (%)	70 (86.4%)
Incomplete or failed occlusion, *n* (%)	8 (9.9%)
Performed secondary treatment (surgery or embolization), *n* (%)	6 (7.4%)
Treatment- or hospital-related complications, *n* (%)	16 (19.8%)

SDAVF, spinal dural arteriovenous fistula; SD, standard deviation; BMI, body mass index; MRI, magnetic resonance imaging; MRA, magnetic resonance angiography; DSA, digital subtraction angiography.

#### 3.1.2 Comorbidity

A total of 31 patients had vascular disease, coronary heart disease or stroke (38.3%), 57 hypertension (70.4%), 23 hyperlipidaemia (28.4%), 27 degenerative spine disease (33.3%), 2 vertebral fractures (2.5%), 1 rheumatic disease (1.2%), 21 used anticoagulant (25.9%), 15 received corticosteroids (18.5%), and 19 were obese with a BMI over 30 kg/m^2^ (23.5%).

#### 3.1.3 Diagnostics

All patients had undergone preoperative spinal angiography to confirm the suspected diagnosis of SDAVF after the initial MRI or MRA examination revealed suspicious findings such as flow voids and myelopathy (81, 100%). Out of 81 patients, 72 underwent MRI/MRA postoperatively (80.2%), while 63 patients received postoperative spinal angiography (77.8%).

#### 3.1.4 Treatments

A total of 70 patients were treated surgically (86.4%) and 11 endovascularly (13.6%). Incomplete or failed occlusion of the fistula occurred in 8 patients (9.9%), with 6 patients (7.4%) undergoing further treatment either surgically or endovascularly. Treatment- or hospital-related complications were observed in 16 patients (19.8%), including five urinary tract infections (6.2%), two deep vein thrombosis (DVT) (2.5%), two strokes (2.5%), one stroke and peritonitis (1.2%), one DVT (1.2%), one DVT and pulmonary embolism (PE) (1.2%), one PE with death (1.2%), one microcatheter rupture (1.2%), one deep wound healing disorder with revision surgery (1.2%), one cerebrospinal fluid fistula (1.2%). Neurological deterioration was considered separately in the clinical outcome.

#### 3.1.5 Treatment comparison

Incomplete or failed occlusions were more common in endovascularly treated patients than in surgically treated patients (surgical: 3/70, 4.3% vs. endovascular: 5/11, 45.5%, *p* < 0.001). In the endovascularly treated patients, this led to four repeat treatments, of which three were treated surgically, one was treated again endovascularly, and one patient refused treatment. In the surgically treated patients, one patient was treated surgically again, one was treated endovascularly, and the third refused further treatment.

The treatment- or hospital-related complications were similar in both groups (surgical: 15/70, 21.4% vs. endovascular: 1/11, 9.1%, *p* = 0.542). The only complication in the endovascular patients was a rupture of the microcatheter. When the microcatheter was withdrawn after application of the Histoacryl-Lipiodol mixture, the catheter was fixed in the vessel by the Histoacryl, and after rupture, a microcatheter residue remained in the vessel, extending from the fistula point to the right superficial femoral artery. The patient received platelet aggregation inhibition with 100 mg ASA for 6 months to prevent thromboembolic complications from the intravascular catheter residue.

The three surgical cases with failed closure were due to mislocalization of the fistula and failure to perform indocyanine green fluorescein angiography (ICGFA) or intraoperative microvascular Doppler sonography (IOMVDS), as the fistula point was found either cranial or caudal to the approach on retreatment. Recanalization was the reason for embolization. One of them was the patient with a catheter rupture who refused further treatment. Overall, 5 of the 8 patients with incomplete or failed closure had a lumbosacral fistula point, five at the S1 level and one at the L5 level.

### 3.2 First symptom in patients with SDAVF

**Figure 2:**
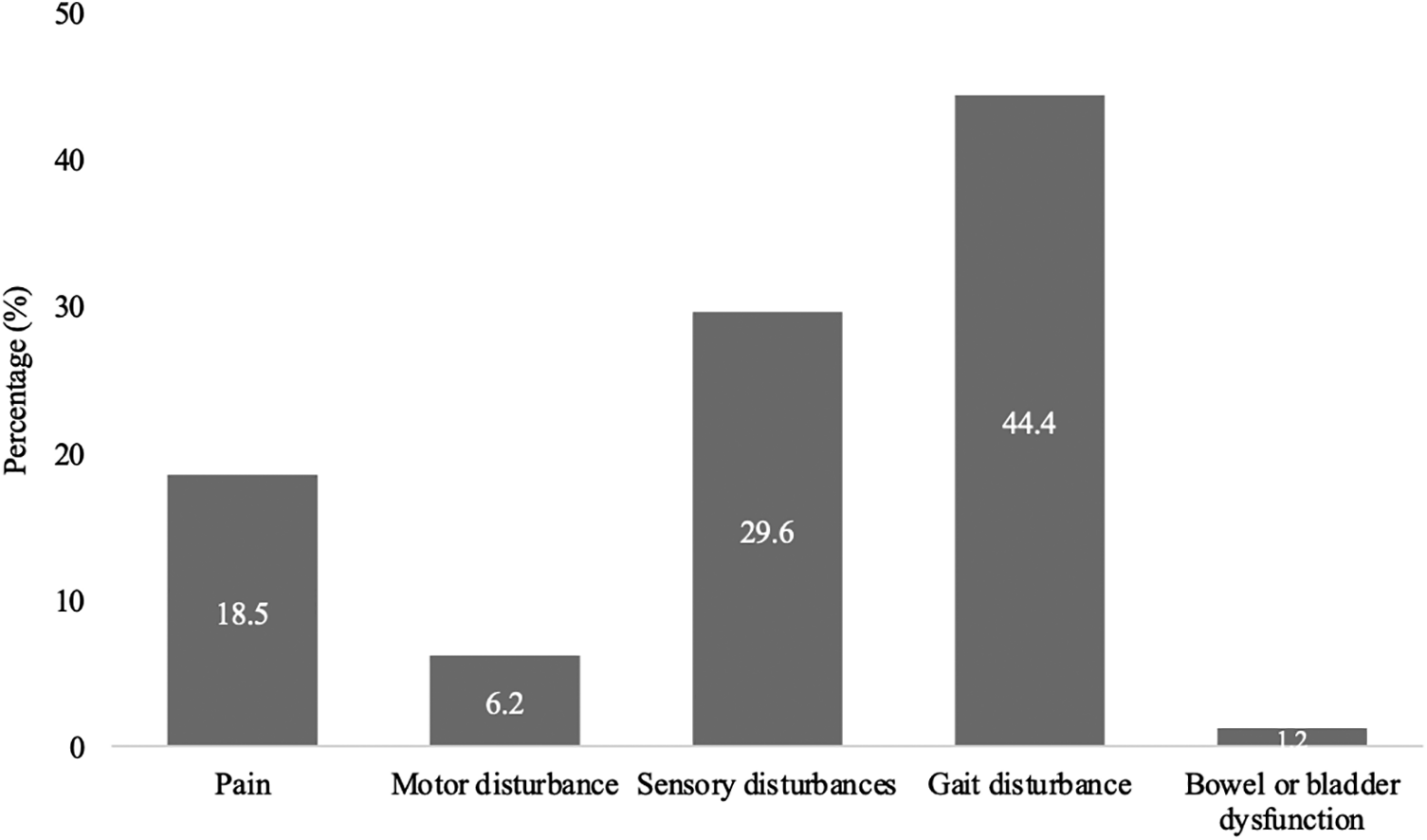
First symptom occurring in SDAVF. This figure shows first symptom occurring in patients with spinal dural arteriovenous fistula (SDAVF).

The most common first symptom in the 81 patients was gait disturbance (36, 44.4%), followed by sensory disturbances such as paraesthesia, hypesthesia, and anaesthesia (24, 29.6%), pain (15, 18.5%), extremity paresis (5; 6.2%) and bowel or bladder dysfunction (1, 1.2%) (Figure 2).

### 3.3 Fistula features

The location of the fistula point was most common in the lower thoracic region (T6–T12; *n* = 36, 44.5%), followed by the lumbar region (L1–L5; *n* = 23, 28.4%), the upper thoracic region (T1–T6; *n* = 12, 14.8%), the sacral region (from S1, *n* = 6, 7.4%) and the cervical region (C1–C8; *n* = 4, 4.9%). Fistula point was on the right side in 46 patients (56.8%) and left in 33 patients (40.7%), with two patients having fistulas on both sides (2.5%) (Table 2).

**Table 2:** Characteristics of fistula.

Side of fistula	
Right, *n* (%)	46 (56.8%)
Left, *n* (%)	33 (40.7%)
Both sides, *n* (%)	2 (2.5%)
Location of fistula point	
Cervical, *n* (%)	4 (4.9%)
Upper thoracic, *n* (%)	12 (14.8%)
Lower thoracic, *n* (%)	36 (44.5%)
Lumbar, *n* (%)	23 (28.4%)
Sacral, *n* (%)	6 (7.4%)

### 3.4 Characteristics of surgical and endovascular treatment

Among the 70 patients treated surgically, single-level laminectomy was the most common approach performed by the surgeon in 31 patients (44.3%), followed by single-level hemilaminectomy in 28 patients (40.0%) and unilateral interlaminar fenestration in 11 patients (15.7%). The average duration of surgery was 138.6 ± 65.7 min. The most common surgical strategy was coagulation and transection (36, 51.4%), followed by a combined strategy of clipping, coagulation, ligation, transection, and clip removal (34, 48.6%). ICGFA was used in 21 patients (30.0%), while IOMVDS was used in 41 patients (58.6%) (Table 3).

**Table 3:** Features of surgical and endovascular treatment.

Surgical (*n* = 70)	
Approach	
Single-level laminectomy, *n* (%)	31 (44.3%)
Single-level hemilaminectomy, *n* (%)	28 (40.0%)
Unilateral interlaminar fenestration, *n* (%)	11 (15.7%)
Duration of operation, mean ± SD (minutes)	138.6 ± 65.7
Surgical techniques	
Coagulation and transection	36 (51.4%)
Combined (clipping, coagulation, ligation, transection, and clip removal), *n* (%)	34 (48.6%)
Use of indocyanine green fluorescein angiography (ICGFA)	21 (30.0%)
Use of intraoperative microvascular doppler sonography (IOMVDS)	41 (58.6%)
Endovascular (*n* = 11)	
Use of histoacryl, *n* (%)	7 (63.6%)
Use of squid, *n* (%)	1 (9.1%)
Use of onyx, *n* (%)	3 (27.3%)

SD, standard deviation; BMI, body mass index.

The endovascular embolization was most frequently performed with Histoacryl (7, 63.6%), followed by Onyx (3, 27.3%), and Squid (1, 9.1%).

### 3.5 Clinical outcomes

#### 3.5.1 Pain

**Figure 3:**
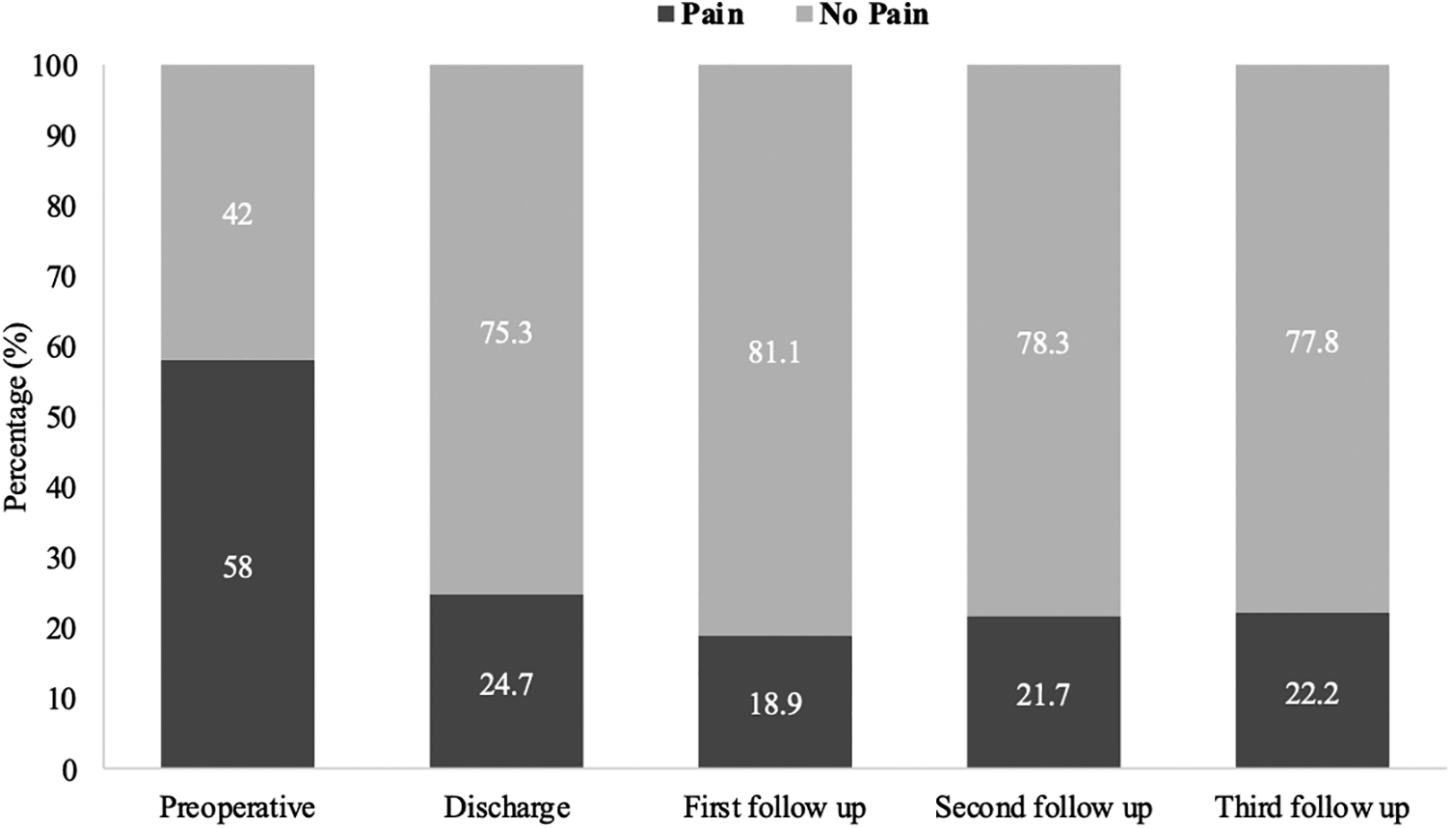
Short and long-term outcomes of pain in patients with SDAVF. This figure shows short- and long-term outcomes of pain in patients with spinal dural arteriovenous fistula (SDAVF).

Back pain or radiculopathy was observed in 58.0% of patients (47/81) with confirmed SDAVF preoperatively. At the time of hospital discharge, 24.7% of patients (20/81) still had pain, 18.9% (14/74) at the first follow-up, 21.7% (15/69) at the second follow-up, and 22.2% (6/27) at the third follow-up (*p* < 0.001) (Figure 3).

#### 3.5.2 Sensory disturbances

**Figure 4:**
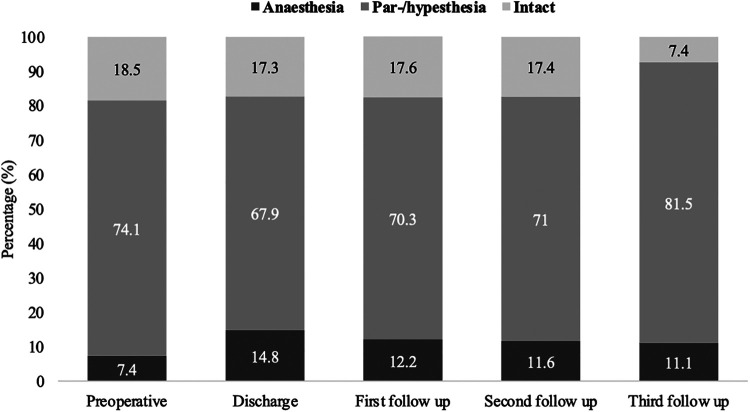
Short- and long-term outcomes of sensory disturbances in patients with SDAVF. This figure shows short- and long-term outcomes of sensory disturbances in patients with spinal dural arteriovenous fistula (SDAVF).

Sensory disturbances such as para-/ or hypesthesia were observed preoperatively in 74.1% of patients (60/81) with confirmed SDAVF. At the time of hospital discharge, 67.9% of patients (55/81) still had para-/or hypesthesia, 70.3% (52/74) at the first follow-up, 71.0% (49/69) at the second follow-up and 81.5% (22/27) at the third follow-up (Figure 4).

Considering patients with intact sensory vs. patients with sensory disturbances over the above intervals, no significant difference was found (preoperative: 15/81, 18.5%; hospital discharge: 14/81, 17.3%; first follow-up: 13/74, 17.6%; second follow-up: 12/69, 17.4%, third follow-up: 2/27, 7.4%) (*p* = 0.681).

Anaesthesia was observed in 7.4% (6/81) of patients with confirmed SDAVF at admission, 14.8% (12/81) at hospital discharge, 122.2% (9/74) at the first follow-up, 11.6% (8/69) at the second follow-up, and 11.1% (3/27) at the third follow-up.

#### 3.5.3 Motor score based on the American spinal injury association grading system (ASIA-Ms)

**Figure 5:**
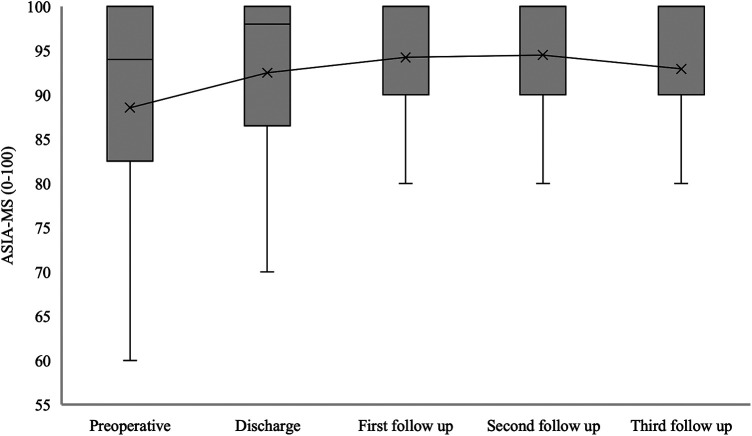
Short- and long-term outcomes of American spinal injury association motor score (ASIA-MS) in patients with SDAVF. This figure shows short- and long-term outcomes of ASIA-MS in patients with spinal dural arteriovenous fistula (SDAVF). ASIA, American Spinal Injury Association; MS, motor score.

The median of ASIA-MS for patients with confirmed SDAVF was 94 [82.5–100] at admission, 98 [86.5–100] at hospital discharge, 100 [90–100] at the first follow-up, 100 [90–100] at the second follow-up, and 100 [90–100] at the third follow-up (*p* = 0.019) (Figure 5).

#### 3.5.4 Modified aminoff-logue scale of disability (mALS)

**Figure 6:**
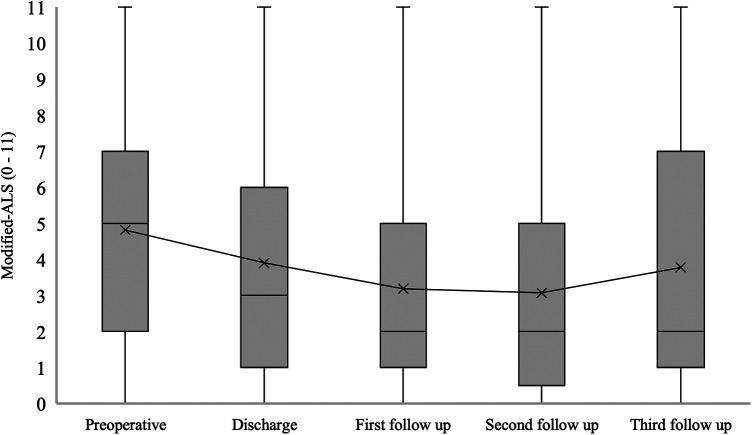
Short- and long-term outcomes of modified aminoff-logue scale (mALS). This figure shows short- and long-term outcomes of modified Aminoff-Logue Scale (mALS) in patients with spinal dural arteriovenous fistula (SDAVF).

The median mALS for patients with confirmed SDAVF was 5 [2–7] at admission, 3 [1–6] at hospital discharge, 2 [1–5] at the first follow-up, 2 [0.5–5] at the second follow-up and 2 [1–7] at the third follow-up (*p* = 0.006) (Figure 6).

**Figure 7:**
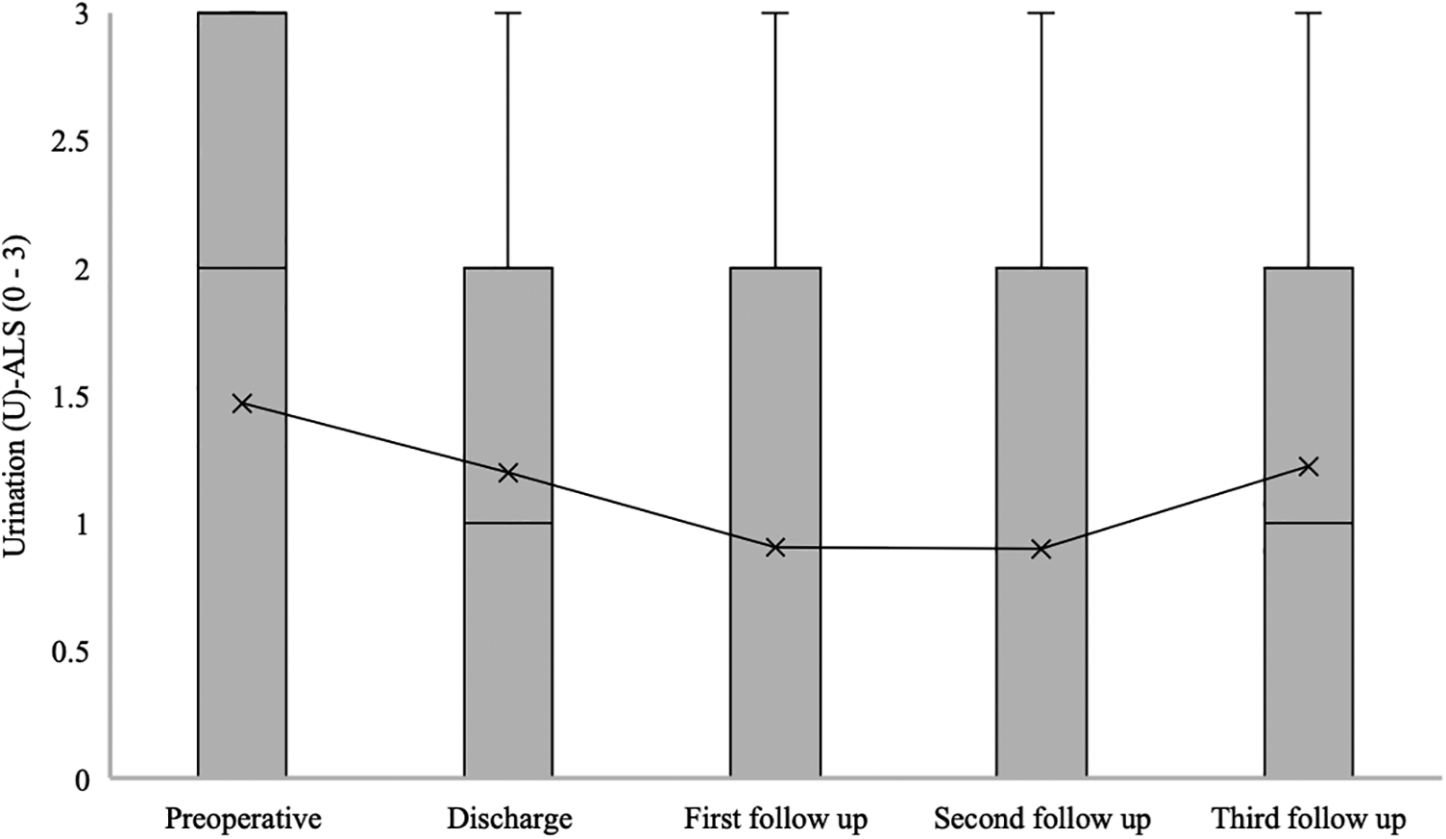
Short- and long-term outcomes of urination (U) aminoff-logue scale (U-ALS). This figure shows short- and long-term outcomes of urination (U) Aminoff-Logue Scale (U-ALS) in patients with spinal dural arteriovenous fistula (SDAVF).

The median urination (U) at ALS for patients with confirmed SDAVF was 2 [0–3] at admission, 1 [0–2] at hospital discharge, 0 [0–2] at the first follow-up, 0 [0–2] at the second follow-up, and 0 [0–1] at the third follow-up (*p* = 0.021) (Figure 7).

**Figure 8:**
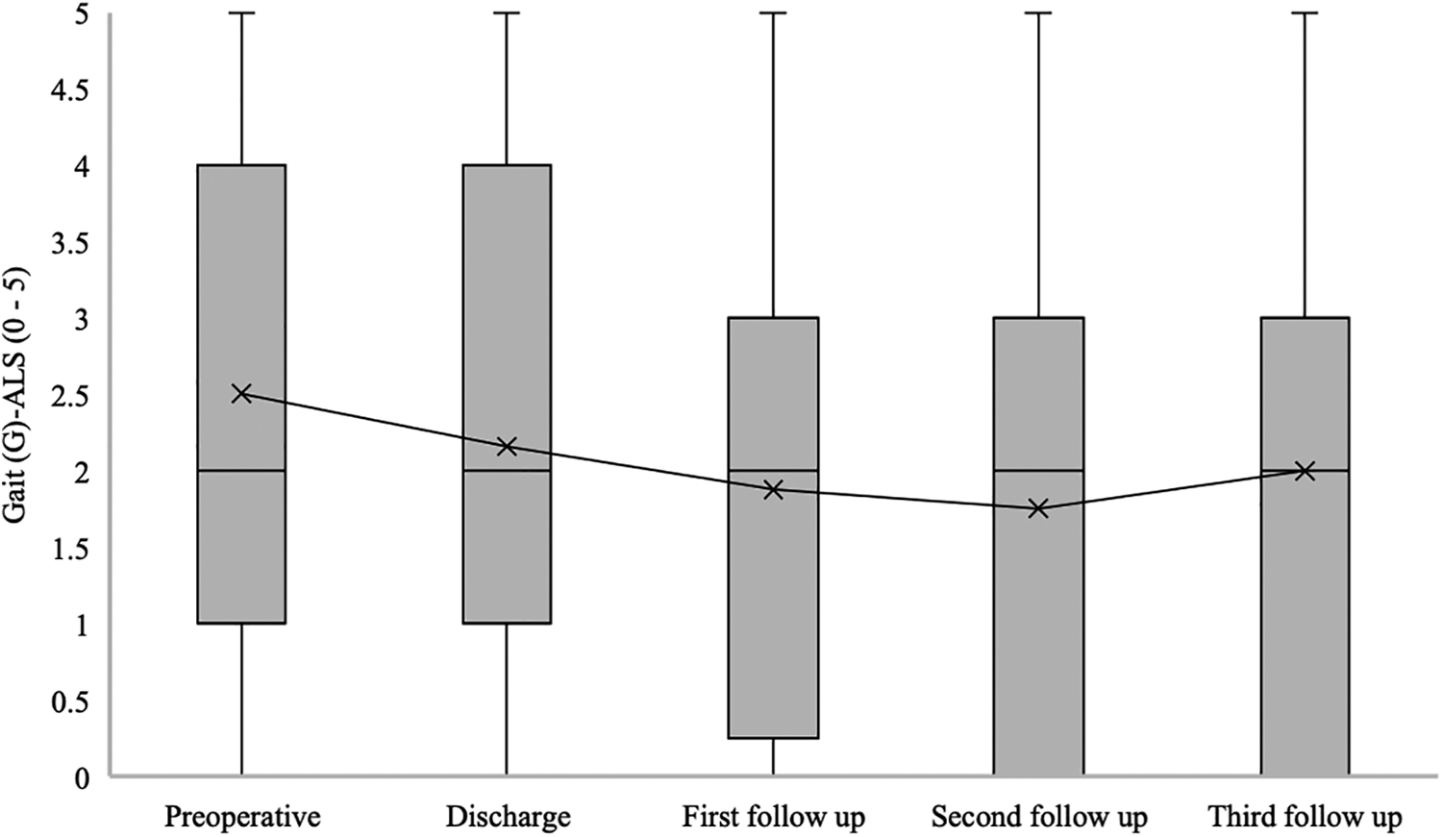
Short- and long-term outcomes of gait (G) aminoff-logue scale (G-ALS). This figure shows short- and long-term outcomes of gait (G) Aminoff-Logue Scale (G-ALS) in patients with spinal dural arteriovenous fistula (SDAVF).

The median gait disturbance (G) at ALS for patients with confirmed SDAVF was 2 [1–4] at admission, 2 [1–4] at hospital discharge, 2 [0–3] at the first follow-up, 2 [0–3] at the second follow-up, and 2 [0–3] at the third follow-up (*p* = 0.04) (Figure 8).

**Figure 9:**
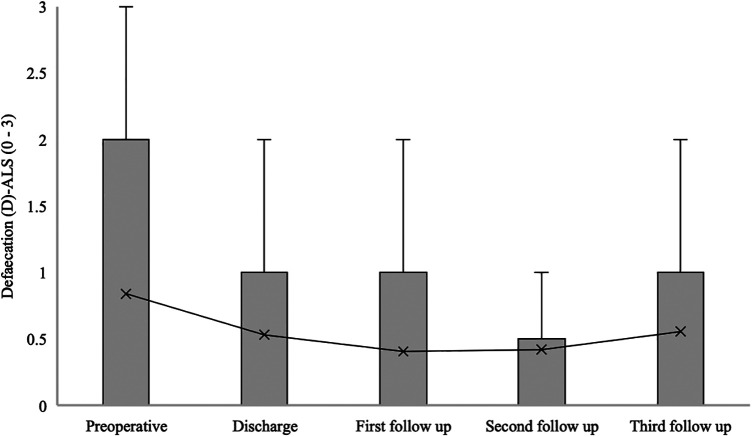
Short- and long-term outcomes of defaecation (D) aminoff-logue scale (D-ALS). This figure shows short- and long-term outcomes of defaecation (D) Aminoff-Logue Scale (D-ALS) in patients with spinal dural arteriovenous fistula (SDAVF).

The median defaecation (D) at ALS for patients with confirmed SDAVF was 0 [0–2] at admission, 1 [0–1] at hospital discharge, 0 [0–1] at the first follow-up, 0 [0–0.5] at the second follow-up, and 0 [0–1] at the third follow-up (*p* = 0.024) (Figure 9).

## 4 Discussion

The key message from this study and our 22 years of experience with SDAVF is that surgical and endovascular treatment represents a safe procedure that leads to a significant decrease in pain, improvement in gait, motor dysfunction and bowel and bladder dysfunction in the short and long term.

In our cohort, SDAVF occurred predominantly in men (3 men to 1 woman) and in the 6 decades of life, as reported in previous studies (6). In our cohort, the diagnosis of SDAVF was delayed on average by 12 months, only one third of the patients had acute progressive symptoms of less than 3 months. After diagnosis, it took 24 days to receive endovascular or surgical treatment. This explains how difficult the diagnosis of SDAVF can be, and also means that some patients had a long period of symptomatic myelopathy before definitive treatment, which also correlates with a poorer clinical outcome, as reported by Roland et al. (21).

Seventy-five percent of our patients had arterial hypertension and 40% had vascular disease. This percentage of patients is elevated. As far as we know, it has not yet been investigated whether there is a connection between these diseases. Surgical treatment was performed in the majority of our patients, which is in line with the current recommendation (17, 18). Our treatment- or hospital-related complications occurred in 19.8% of patients, one patient died due to PE. The main complications were embolisms in bedridden patients and urinary tract infections in patients with urinary incontinence.

Gait disturbance seems to be the main and most common first symptom in SDAVF in our cohort, which can be explained by the vascular myelopathy. Followed by sensory disturbance and pain. Our cohort has shown that one half of SDAVFs are localized in the lower thoracic spine and that there is no side preference. Numerous studies claim that the pathology occurs predominantly on the left side (9, 10).

Between 2002 and 2017, a single-level laminectomy was performed in the majority of cases (45%) to access the SDAVF. The reason why the surgeon chose single-level laminectomy was the better surgical visibility and the ease of performing a watertight dural suture in older and partially obese patients. A single-level hemilaminectomy was the standard approach in our hospital and was performed in about 40% of patients. In 15% of patients, even unilateral interlaminar fenestration was successfully performed. This approach also appears to be sufficient. Overall, of the 70 patients treated surgically, only one patient developed a cerebrospinal fluid fistula, and one patient developed a deep wound healing disorder with revision surgery, so there was no significant difference between the three approaches. The literature is not unanimous in this respect, with some authors still favouring single-level laminectomy (1, 18). We prefer to perform a hemilaminectomy in rigid or semi-rigid spinal segments, although a laminectomy can be performed where required for dural sutures and better visibility. We recommend a unilateral interlaminar fenestration for a junctional and mobile spinal segment.

ICGFA or IOMVDS was used in most cases (62/70). We currently use ICGFA as the standard technique in our hospital. No significant difference was found between the two surgical techniques (coagulation and transection vs. combined procedure). The operation lasted on average about 2 h and has a low rate of complications. We mainly used Histoacryl for endovascular treatment. The difference between the used and the available embolization material has to be investigated in a multicentre prospective study.

In previous studies, patients were clinically examined in relation to mALS; however, sensory and motor disturbances and pain were not considered. Postoperatively, pain symptoms disappeared completely in 30% of patients, while 42% had no pain preoperatively. In contrast, we found no significant difference in sensory disturbances. The majority of patients maintained hyp-, para- or anaesthesia postoperatively and in the further course. The median ASIA-MS increased significantly from 94 to 100.

In our study, a significant improvement in mALS, Gait-ALS, Urination-ALS and Defaecation-ALS was observed in the further course of the disease (discharge, first, second and third follow-up) compared to the preoperative assessment. This observation was noted in previous studies, some of which found no significant improvement in urination-ALS (21, 23, 24).

### 4.1 Limitations and strengths of this study

The monocentric, retrospective nature of our analysis, the long inclusion interval, and the limited number of SDAVF patients treated with embolization (11 patients) might reduce the external validity of our study. Furthermore, our analysis could be affected by a possible selection bias due to our treatment flow charts, as our experience has been to favor surgery over embolization. Nevertheless, our cohort analysis is based on a 20-year treatment period of SDAVF in a large university neurosurgery center, suggesting a high internal validity of our study. Therefore, our observations may be useful to understand the clinical and radiologic characteristics of SDAVF.

## 5 Conclusions

Our retrospective cohort study and 22 years of experience with SDAVF demonstrate safe therapy with significantly reduce in pain, improvement in gait disturbance, motor deficit, bowel, and bladder dysfunction in the short and long term, but not in sensory disturbances.

The type of surgical approach (hemi-, laminectomy, or unilateral interlaminar fenestration), the surgical techniques (coagulation and transection or combined technique) and the methods used to confirm the occlusion intraoperatively (ICGFA or IOMVDS) do not play a role in the outcome. Nevertheless, we recommend unilateral interlaminar fenestration, ICGFA use, and combined technique. A prospective multicenter study with different embolization materials should be investigated.

## Data Availability

The original contributions presented in the study are included in the article/Supplementary Material, further inquiries can be directed to the corresponding author.
